# Changes in blood parameters in spontaneous preterm birth during pregnancy: An observational study

**DOI:** 10.1097/MD.0000000000044524

**Published:** 2025-09-12

**Authors:** Manhua Zhen, Yanfei Zeng, Yuhua Song, Chenggang Yang, Wen Ai

**Affiliations:** a Department of Obstetrics and Gynecology, Foshan Fosun Chancheng Hospital, Foshan, Guangdong Province, China.

**Keywords:** complete blood count, monocyte, pregnancy, spontaneous preterm birth, white blood cell

## Abstract

Spontaneous preterm birth (sPTB) accounts for two-thirds of preterm births and leads to morbidity and death in newborns under 5 years of age. This study aimed to examine the changes in complete blood count parameters in pregnant women with sPTB during pregnancy. This was a retrospective case-control study, with 138 sPTB cases and 138 age- and delivery date-matched healthy controls. Baseline data and the complete blood count parameters examined during the 1st, 2nd, and 3rd trimesters of all the participants were recorded. The *χ*^2^ test, *t*-test, and multivariate linear regression analysis methods were used to analyze the changes in parameters between the 2 groups at 3 time points. The gestational age at delivery was shorter and the birth weight was lower in the sPTB group, compared with the term birth group. The nulliparity and male sex distribution, maternal age, and gestational age at the 1st, 2nd, and 3rd assessments were balanced between the 2 groups. White blood cell and monocyte counts increased throughout pregnancy in both groups, and the sPTB group was consistently higher than the term group. The current findings demonstrated that blood inflammation- and infection-related parameters, such as white blood cell and monocyte counts, changed significantly during pregnancy in women with sPTB. This study may have important implications for the diagnostic and therapeutic management of sPTB.

## 1. Introduction

Preterm birth is defined as a birth under 37 weeks of gestational age and is the leading cause of morbidity and death in newborns under 5 years of age.^[1–3]^ The composite rate of neonatal morbidity of premature infants is 6 times higher than that of infants born at term.^[4]^ The incidence of preterm birth varies among geographic location and socioeconomic status. Reviews report escalating rates of preterm birth worldwide, which are as high as 10% for preterm birth, 8% for very (32–28 weeks) preterm, 5% for extreme (<28 weeks) preterm, and 7% for spontaneous preterm birth (sPTB), 5% for therapeutic preterm birth.^[5,6]^ Most preterm births, roughly two thirds, occur spontaneously, that is, they do not correspond with symptoms that call for the medical initiation of preterm birth.^[7]^ Although several factors, such as maternal age, family cognitions, ethnicity, and sexual intercourse during pregnancy are associated with an increased risk of sPTB, infection and inflammation are the well-known trigger.^[8–11]^

Parameters, especially reflecting inflammatory events, from complete blood count, have been prioritized by many researchers in recent years.^[12]^ Previous studies have observed abnormal levels of complete blood count parameters in pregnant women with sPTB at different times of pregnancy. Pregnant women with sPTB have exhibited increased white blood cell (WBC), platelet, lymphocyte, monocyte, and lymphocyte–monocyte ratio (LMR), reduced platelet–lymphocyte ratio (PLR) and neutrophil–lymphocyte ratio (NLR) in the 1st trimester of pregnancy,^[13]^ increased WBC, NLR, and PLR in the 2nd trimester of pregnancy,^[14–16]^ and increased leukocyte, neutrophil, and NLR, reduced lymphocyte in the 3rd trimester.^[17]^ Despite the results of studies showing the aberrant complete blood count parameters in pregnant women with sPTB, few studies have particularly examined the changes in blood parameters in sPTB during pregnancy. The exact changes in parameters during pregnancy remain elusive.

Given the strong association between inflammation and sPTB, routine blood parameters that reflect inflammatory processes have emerged as promising biomarkers for predicting and understanding sPTB. Parameters such as WBC, LMR, PLR, and NLR are easily accessible, cost-effective, and widely used in clinical practice. These markers not only provide insights into systemic inflammatory states but also offer potential clues about the underlying mechanisms driving sPTB. In this study, we retrospectively examined the changes in routine blood parameters, such as hemoglobin, WBC count, platelet, neutrophil, lymphocyte, monocyte, PLR, NLR, and LMR in sPTB during pregnancy. This study may have important implications for the diagnostic and therapeutic management of sPTB.

## 2. Methods

A total of 276 participants with sPTB at our hospital were enrolled in this study between January and December 2023. The study was approved by the Ethics Committee of our hospital (FFCH-MEC-2020-029) and every participant gave their informed consent.

Any delivery that occurred earlier than 37 weeks + 0 days gestation was considered preterm. Any preterm birth, including preterm premature ruptured membranes, preterm labor and fetal membrane prolapse, was defined as sPTB. Iatrogenic preterm delivery or medically indicated (such as for gestational diabetes mellitus, preeclampsia, multiple gestations, previous history of preterm delivery, etc) were excluded. Meanwhile, the participants with acute infection (fever, pain, or vaginal discharge), acute or chronic inflammatory diseases, hematopoietic system disorders, malignancies, and other systemic diseases were also excluded in this study. These conditions were identified through a combination of clinical records, laboratory diagnostic tests, and physical examinations to ensure accurate and transparent exclusion criteria.^[18–20]^ Gestational age has been established by combining 1st trimester ultrasonography with the last menstrual period.^[21,22]^ The control group recruited women who were 37 to 41 weeks pregnant and had no history of pregnancy-related issues, while also controlling for similar age and delivery dates.

The maternal age, parity, gestational age at delivery, birth weight, and results of complete blood count in maternal serum at 1st trimester between 8 0/7 weeks and 13 6/7 weeks of gestation, 2nd trimester between 14 0/7 weeks and 27 6/7 weeks of gestation, and 3rd trimester between 28 0/7 weeks and 36 6/7 weeks of gestation were recorded from the hospital’s medical records and electronic laboratory system. The Coulter LH 780 device (Beckman Coulter, Brea), as well as its quality control products and supporting reagents, were employed for complete blood counts. If multiple data was available for the same pregnant woman within a same point, the sample closest to 10 gestation weeks, 24 gestation weeks, and 35 gestation weeks were utilized. Hemoglobin, WBC, platelet, neutrophil, lymphocyte, and monocyte counts were directly extracted, and the PLR (platelet-to-lymphocyte), NLR (neutrophil-to-lymphocyte), and LMR (lymphocyte-to-monocyte) were calculated using the counts of the platelet, lymphocyte, neutrophil, and monocyte.

### 2.1. Statistical analysis

Statistical analyses were performed using R software version 4.3.1 (The R Foundation, Hefei, China).^[23,24]^ Data were presented as frequencies and percentages (for categorical data) and mean ± standard deviation (for continuous data). The *χ*^*2*^ and *t*-test analyses were used to analyze categorical and continuous variables, respectively. Adjusted multivariate linear regression analysis, accounting for potential confounders such as age and gestational age at assessment, was conducted using the lm() function to ensure the stability and robustness of the results. Subgroup analyses were conducted based on the type of preterm birth: late preterm (between 34 0/7 weeks and 36 6/7 weeks of gestation), moderate preterm (between 32 0/7 weeks and 33 6/7 weeks of gestation), and very preterm (before 32 weeks of gestation).^[25,26]^ Additional packages, including dplyr for data manipulation and ggplot2 for visualization, were utilized where necessary. A *P* value < .05 was considered as statistically significant.

This is a case-control study in which multivariable regression analysis was used to compare the 2 groups. Six parameters were investigated in this study. Observational analyses were performed for each parameter with sample sizes ranging from 15 to 30.^[27,28]^ A total of 90 participants per group were initially required for the study. However, accounting for potential missing values in some variables, a final sample size of 138 participants per group was deemed sufficient to ensure robust and reliable results.

## 3. Results

**Figure 1.**
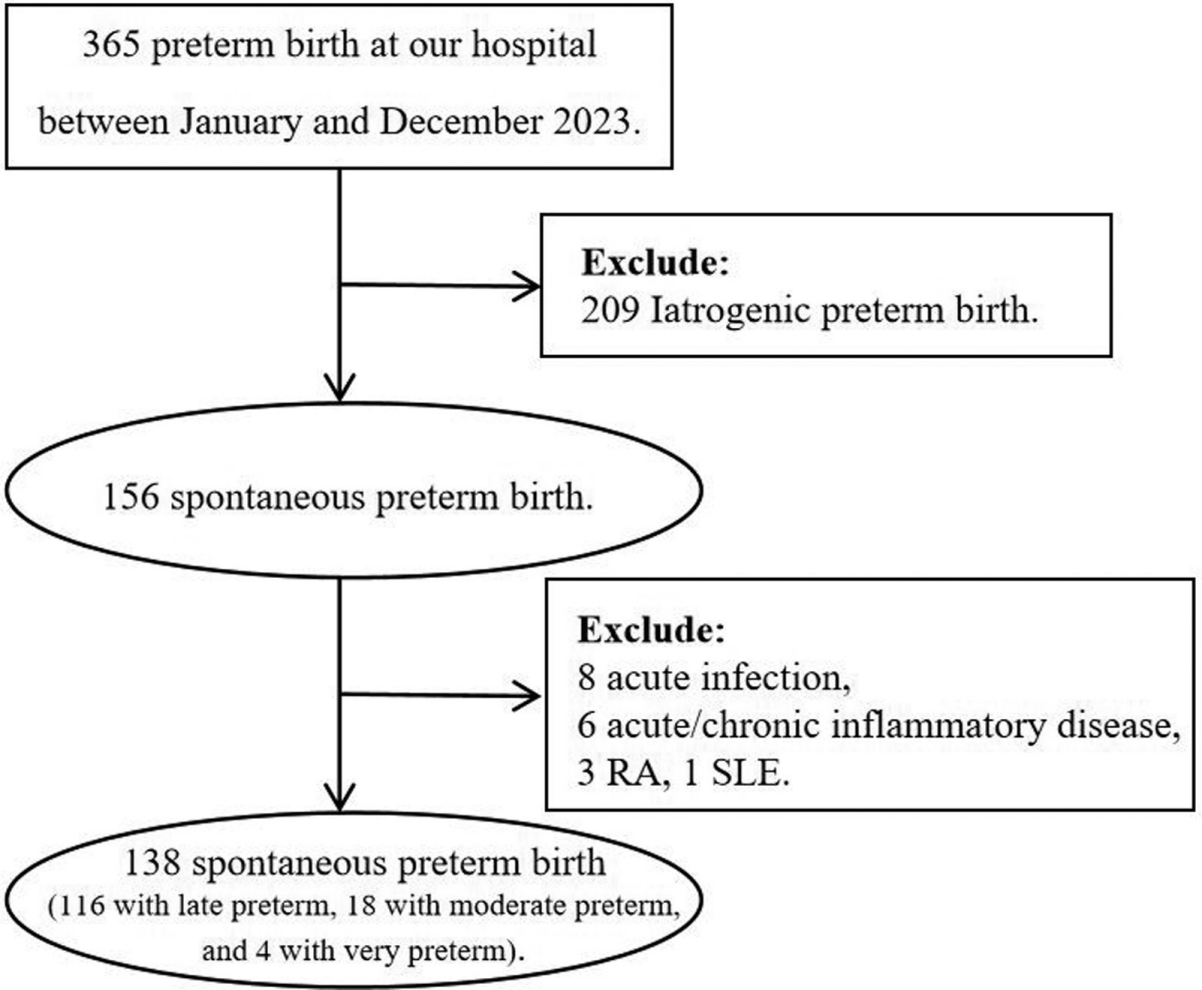
Flow chart of the study participants.

There are 276 participants, including 138 with sPTB (116 with late preterm, 18 with moderate preterm, and 4 with very preterm) and 138 matched term births included in this study. Figure 1 presents a flowchart of the inclusion and exclusion data. The gestational age at the 1st, 2nd, and 3rd assessments was 10.71 ± 6.11, 24.73 ± 3.04, and 35.05 ± 1.42, respectively. The gestational age at delivery was smaller, and the birth weight was lower in the sPTB group, compared with the term birth group (35.38 ± 1.63 vs 39.31 ± 0.93, *P* = .001; 2447.50 ± 510.58 vs 3336.30 ± 411.05, *P* = .001; respectively). The nulliparity and male sex distribution, maternal age, and gestational age at the 1st, 2nd, and 3rd assessment were balanced between the 2 groups (Table 1).

**Table 1:** Characteristics and results of the study groups.

	Preterm delivery (n = 138)	Term delivery (n = 138)	*t/χ* ^*2*^	*P*	*t* *	*P* *
Maternal age (yr)	29.62 ± 4.34	29.91 ± 3.12	0.621	.535	–	–
Gestational age at delivery (wk)	35.38 ± 1.63	39.31 ± 0.93	24.655	.001	–	–
Birth weight (g)	2447.50 ± 510.58	3336.30 ± 411.05	15.929	.001	–	–
Nulliparity	72 (52.2%)	71 (51.4%)	0.015	.999	–	–
Male sex	76 (55.1%)	67 (48.6%)	1.175	.278	–	–
Gestational age at 1st assessment (wk)	10.61 ± 4.56	10.81 ± 7.36	0.271	.787	–	–
Gestational age at 2nd assessment (wk)	24.79 ± 3.69	24.67 ± 2.22	0.336	.737	–	–
Gestational age at 3rd assessment (wk)	35.03 ± 1.88	35.07 ± 0.72	0.230	.818	–	–
First assessment						
Hemoglobin (g/L)	122.60 ± 11.41	122.07 ± 9.79	0.419	.675	0.365	.715
WBC count (*10^9^/L)	9.77 ± 2.20	7.48 ± 1.58	9.957	.001	10.036	.001
Platelet (*10^9^/L)	267.72 ± 61.44	251.72 ± 47.69	2.417	.016	2.383	.018
Neutrophil (*10^9^/L)	6.09 ± 1.82	5.83 ± 1.68	1.227	.221	1.368	.172
Lymphocyte (*10^9^/L)	2.56 ± 0.58	1.73 ± 0.55	12.285	.001	12.327	.001
Monocyte (*10^9^/L)	0.54 ± 0.17	0.48 ± 0.15	3.092	.002	3.048	.001
PLR	108.42 ± 30.75	158.43 ± 53.45	9.527	.001	9.485	.001
NLR	2.46 ± 0.82	3.71 ± 1.61	8.185	.001	8.354	.001
LMR	5.29 ± 2.49	3.96 ± 2.00	4.885	.001	4.890	.001
Second assessment						
Hemoglobin (g/L)	130.57 ± 5.98	112.44 ± 8.44	1.687	.094	1.678	.095
WBC count (*10^9^/L)	10.27 ± 2.53	8.60 ± 2.17	5.901	.001	5.848	.001
Platelet (*10^9^/L)	249.73 ± 58.24	234.26 ± 51.26	2.342	.020	2.345	.020
Neutrophil (*10^9^/L)	7.44 ± 2.13	7.16 ± 1.82	1.157	.248	1.149	.251
Lymphocyte (*10^9^/L)	1.95 ± 0.53	1.85 ± 0.45	1.765	.079	1.716	.087
Monocyte (*10^9^/L)	0.62 ± 0.20	0.58 ± 0.19	1.790	.075	1.681	.094
PLR	135.63 ± 48.17	132.31 ± 37.01	0.641	.522	0.652	.515
NLR	4.04 ± 1.52	3.98 ± 1.02	0.365	.716	0.386	.699
LMR	3.35 ± 1.08	3.40 ± 0.97	0.382	.703	0.267	.790
Third assessment						
Hemoglobin (g/L)	120.14 ± 75.53	116.13 ± 10.59	0.617	.538	0.597	.551
WBC count (*10^9^/L)	11.51 ± 7.37	9.10 ± 2.25	3.678	.001	3.659	.001
Platelet (*10^9^/L)	243.54 ± 76.11	231.24 ± 50.87	1.579	.116	1.568	.118
Neutrophil (*10^9^/L)	8.64 ± 5.33	6.52 ± 2.07	4.356	.001	4.328	.001
Lymphocyte (*10^9^/L)	1.74 ± 0.58	1.73 ± 0.45	0.291	.771	0.283	.777
Monocyte (*10^9^/L)	0.72 ± 0.28	0.65 ± 0.20	2.317	.021	2.239	.026
PLR	154.88 ± 79.53	141.05 ± 41.12	1.815	.071	1.825	.069
NLR	5.59 ± 4.36	4.05 ± 1.95	3.791	.001	3.786	.001
LMR	2.71 ± 1.28	2.84 ± 0.97	0.963	.336	0.908	.365

LMR = lymphocyte–monocyte ratio, NLR = neutrophil–lymphocyte ratio, PLR = platelet–lymphocyte ratio, WBC = white blood cell.

* Adjusted for age and gestational age at assessment.

For the 1st assessment, the WBC count (9.77 ± 2.20 vs 7.48 ± 1.58, *P* = .001), platelet count (267.72 ± 61.44 vs 251.72 ± 47.69, *P* = .016), lymphocyte count (2.56 ± 0.58 vs 1.73 ± 0.55, *P* = .001), monocyte count (0.54 ± 0.17 vs 0.48 ± 0.15, *P* = .002), NLR (2.46 ± 0.82 vs 3.71 ± 1.61, *P* = .001), and LMR (5.29 ± 2.49 vs 3.96 ± 2.00, *P* = .001) were higher, and the PLR (108.42 ± 30.75 vs 158.43 ± 53.45, *P* = .001) was lower in the sPTB group than in the term group. The hemoglobin and neutrophil levels were balanced between the 2 groups. For the 2nd assessment, the WBC count and platelet count were higher in the sPTB group compared with the term birth group (10.27 ± 2.53 vs 8.60 ± 2.17, *P* = .001; 249.73 ± 58.24 vs 234.26 ± 51.26, *P* = .020; respectively). Other parameters were comparable between the 2 groups. For the 3rd assessment, the WBC count (11.51 ± 7.37 vs 9.10 ± 2.25, *P* = .001), neutrophil count (8.64 ± 5.33 vs 6.52 ± 2.07, *P* = .001), monocyte count (0.72 ± 0.28 vs 0.65 ± 0.20, *P* = .021), PLR (154.88 ± 79.53 vs 141.05 ± 41.12, *P* = .071), and NLR (5.59 ± 4.36 vs 4.05 ± 1.95, *P* = .001) were higher in the sPTB group than in the term group. The hemoglobin, platelet, lymphocyte, and NLR levels were all balanced between the 2 groups. After adjusting for age and gestational age at assessment, the results did not change substantially (Table 1). Subgroup analyses showed that the results for late preterm birth were also consistent with the above (Table 2).

**Table 2:** Characteristics and results of the study in late preterm delivery group.

	Late preterm delivery (n = 116)	Term delivery (n = 116)	*t/χ* ^*2*^	*P*	*t* *	*P* *
Maternal age (yr)	29.56 ± 4.34	29.86 ± 3.24	0.610	.542	–	–
Gestational age at delivery (wk)	35.90 ± 0.88	39.06 ± 0.76	29.171	.001	–	–
Birth weight (g)	2555.12 ± 461.07	3291.63 ± 397.20	13.035	.001	–	–
Nulliparity	58 (50.0%)	57 (49.1%)	0.017	.896	–	–
Male sex	56 (48.3%)	65 (56.0%)	1.399	.237	–	–
Gestational age at 1st assessment (wk)	10.24 ± 4.46	10.81 ± 7.93	0.271	.787	–	–
Gestational age at 2nd assessment (wk)	24.71 ± 3.67	24.65 ± 2.38	0.137	.892	–	–
Gestational age at 3rd assessment (wk)	35.56 ± 1.30	35.48 ± 0.74	0.120	.618	–	–
First assessment						
Hemoglobin (g/L)	122.92 ± 11.25	122.51 ± 9.54	0.302	.763	0.209	.835
WBC count (*10^9^/L)	9.93 ± 2.25	7.43 ± 1.65	9.616	.001	9.790	.001
Platelet (*10^9^/L)	270.86 ± 60.68	252.33 ± 47.87	2.583	.010	2.537	.012
Neutrophil (*10^9^/L)	6.16 ± 1.88	5.77 ± 1.75	1.629	.105	1.902	.058
Lymphocyte (*10^9^/L)	2.61 ± 0.60	1.74 ± 0.55	11.416	.001	11.354	.001
Monocyte (*10^9^/L)	0.55 ± 0.17	0.49 ± 0.15	2.868	.005	2.841	.005
PLR	107.94 ± 30.64	156.87 ± 50.59	8.908	.001	8.822	.001
NLR	2.44 ± 0.83	3.65 ± 1.64	7.083	.001	7.090	.001
LMR	5.29 ± 2.53	3.91 ± 1.90	4.700	.001	4.652	.001
Second assessment						
Hemoglobin (g/L)	134.29 ± 7.15	112.85 ± 8.47	1.680	.096	1.700	.091
WBC count (*10^9^/L)	10.42 ± 2.55	8.68 ± 2.29	5.483	.001	5.443	.001
Platelet (*10^9^/L)	253.58 ± 57.59	236.29 ± 52.43	2.391	.018	2.394	.017
Neutrophil (*10^9^/L)	7.54 ± 2.16	7.25 ± 1.81	1.094	.275	1.098	.273
Lymphocyte (*10^9^/L)	1.99 ± 0.52	1.87 ± 0.46	1.848	.066	1.794	.074
Monocyte (*10^9^/L)	0.63 ± 0.21	0.59 ± 0.20	1.620	.106	1.553	.122
PLR	135.47 ± 48.80	132.41 ± 36.91	0.538	.591	0.562	.575
NLR	4.02 ± 1.49	4.00 ± 0.98	0.105	.917	0.145	.885
LMR	3.36 ± 1.03	3.41 ± 1.02	0.360	.719	0.311	.756
Third assessment						
Hemoglobin (g/L)	122.29 ± 82.15	116.33 ± 10.63	0.776	.439	0.490	.624
WBC count (*10^9^/L)	11.57 ± 7.93	9.16 ± 2.36	3.135	.002	3.512	.001
Platelet (*10^9^/L)	246.60 ± 76.29	233.79 ± 52.10	1.494	.137	1.618	.107
Neutrophil (*10^9^/L)	8.68 ± 5.64	6.57 ± 2.17	3.774	.001	4.395	.001
Lymphocyte (*10^9^/L)	1.76 ± 0.57	1.75 ± 0.47	0.100	.920	0.092	.927
Monocyte (*10^9^/L)	0.72 ± 0.28	0.66 ± 0.21	1.834	.068	2.204	.029
PLR	155.31 ± 79.77	140.88 ± 41.56	1.727	.086	1.936	.054
NLR	5.54 ± 4.44	4.05 ± 2.10	3.281	.001	3.904	.001
LMR	2.77 ± 1.34	2.88 ± 1.02	0.6873	.493	0.879	.380

LMR = lymphocyte–monocyte ratio, NLR = neutrophil–lymphocyte ratio, PLR = platelet–lymphocyte ratio, WBC = white blood cell.

* Adjusted for age and gestational age at assessment.

**Figure 2.**
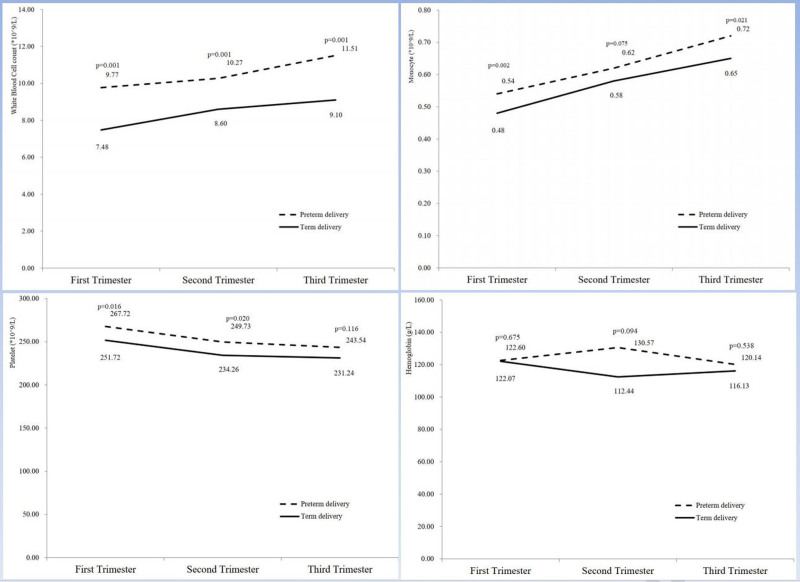
Changes in complete blood count parameters (WBC, monocyte, platelet, and hemoglobin) in pregnant women with spontaneous preterm birth at the 3 different points. WBC = white blood cell.

**Figure 3.**
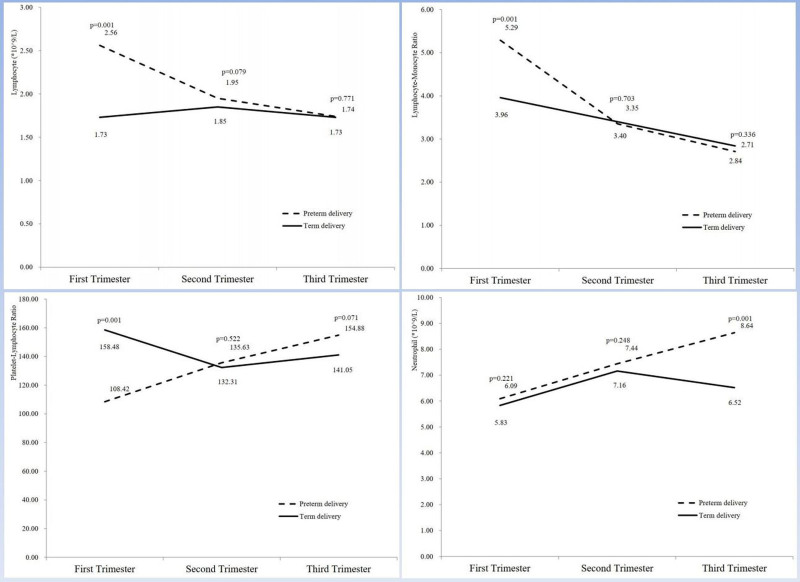
Changes in complete blood count parameters (lymphocyte, LMR, PLR, and neutrophil) in pregnant women with spontaneous preterm birth at the 3 different points. LMR = lymphocyte–monocyte ratio, PLR = platelet–lymphocyte ratio

**Figure 4.**
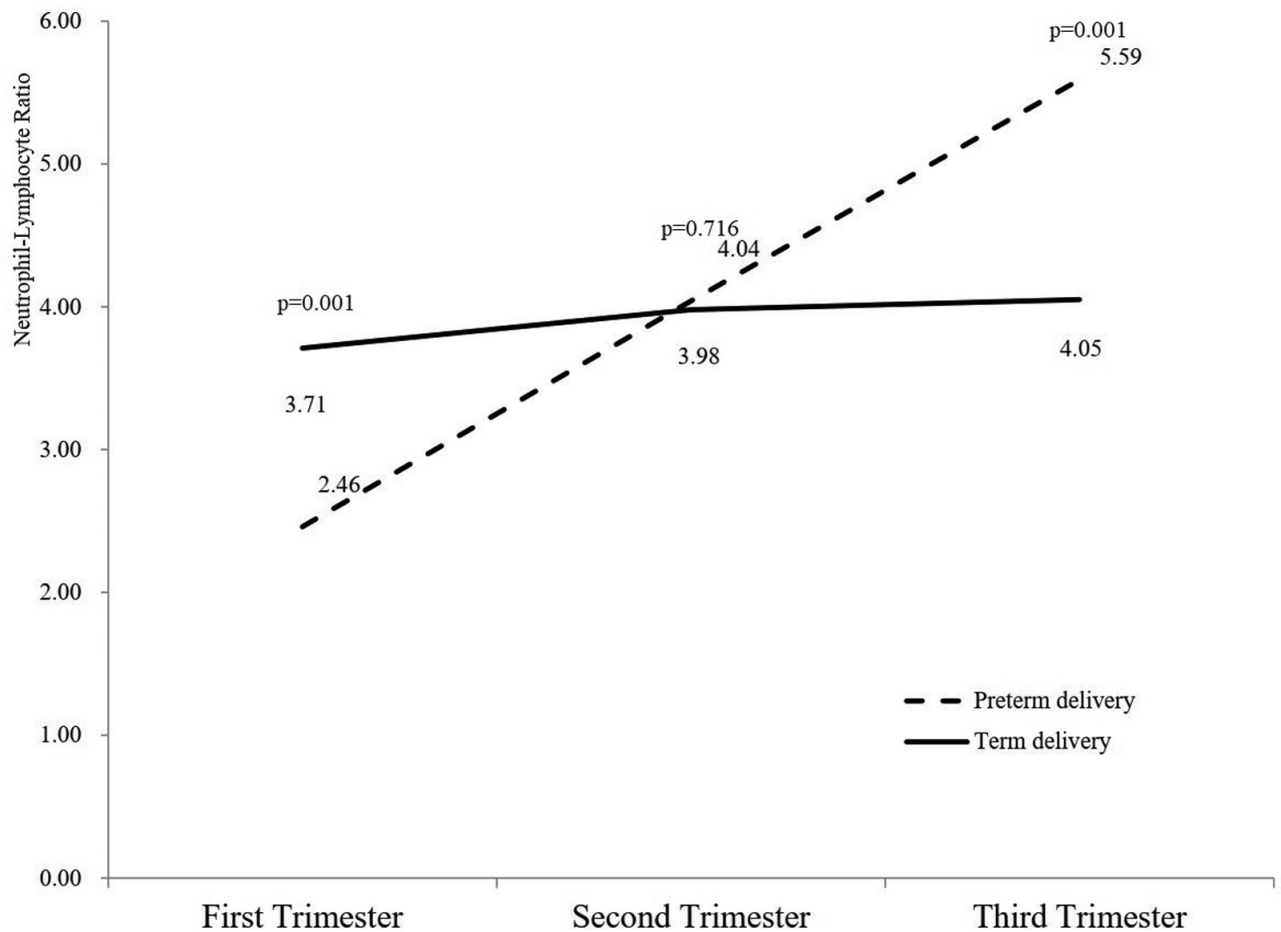
Changes in complete blood count parameters (NLR) in pregnant women with spontaneous preterm birth at the 3 different points. NLR = neutrophil–lymphocyte ratio.

The changes in the above parameters during pregnancy are shown in Figures 2 to 4. WBC and monocyte counts increased throughout pregnancy in both groups, and the sPTB group was consistently higher than the term group. For platelet count, there were differences in the 1st and 2nd trimesters, but the differences disappeared in the 3rd trimester of pregnancy. For hemoglobin, the 2 groups remained comparable throughout pregnancy. Differences in lymphocyte count, LMR, and PLR appeared only in the 1st trimester and then gradually decreased between the 2 groups. For neutrophil count, the difference appeared in the 3rd trimester of pregnancy. For NLR, there was a crossover between the 2 groups during the 2nd trimester.

## 4. Discussion

In the present study, changes in complete blood count parameters, particularly those related to inflammatory and infection events, were examined throughout the course of gestation in sPTB.

Preterm birth could afflict 10% of children born worldwide, and 2 in 3 preterm births occur spontaneously.^[29]^ Acute or chronic intrauterine inflammation or infection are common obstetric complications, which increase the risk of sPTB.^[13]^ Local inflammatory processes have been considered to be the most important pathogenesis of sPTB, and the distinct inflammatory nature of sPTB has been a focus of researchers. In women with sPTB, abnormal levels of certain complete blood count parameters typically arise during pregnancy.^[30,31]^

WBC and monocytes in peripheral blood are sensitive to changes in inflammatory and infection responses.^[32,33]^ Monocytes, key mediators of inflammation, showed similar trends, corroborating earlier findings that monocyte levels are elevated early in pregnancy among women who eventually experience sPTB.^[13]^ Data from previous studies revealed that WBC values are higher during the 2nd trimester of pregnancy in sPTB.^[14,16]^ We previously found that WBC and monocytes during the 1st trimester of pregnancy are significantly higher in sPTB.^[13]^ On the other hand, researchers also found that WBC counts showed no significant differences between full-term pregnancies and preterm births at the 3rd trimester.^[34]^

Levels of WBC and monocytes show changes in systemic inflammatory and infection events.^[35,36]^ In our study, we observed that WBC and monocyte levels increased throughout pregnancy in both the sPTB group and the term control group, with the sPTB group consistently exhibiting higher levels. This finding is consistent with previous research indicating that WBC values tend to rise during pregnancy, particularly in women experiencing sPTB.^[14,16]^ Our results extend these observations by demonstrating a continuous increase from the 1st to the 3rd trimester, highlighting that the inflammatory response may be more pronounced and sustained in women who deliver prematurely. While previous studies have focused on specific time points or isolated comparisons between full-term pregnancies and preterm births,^[34]^ our comprehensive analysis across all 3 trimesters reveals a continuous increase in these markers. This suggests ongoing systemic inflammation or immune dysregulation in women at risk for sPTB. The persistent elevation of monocytes suggests their significant role in the inflammatory processes leading to preterm labor.

The roles of the PLR and LMR as markers of systemic inflammation and immune response are increasingly recognized in the context of sPTB. PLR reflects the balance between platelet activation, which is closely linked to inflammation, and lymphocyte-mediated immune regulation.^[37,38]^ Conversely, LMR highlights the interplay between monocyte-driven inflammation and lymphocyte activity. Abnormal levels of PLR and LMR during pregnancy could serve as early warning signs of heightened inflammatory activity, enabling timely interventions. Previous studies have suggested that aberrant PLR and LMR levels may be associated with adverse pregnancy outcomes, including preterm birth.^[39,40]^

In our study, we observed significant differences in lymphocyte count, LMR, and PLR specifically during the 1st trimester between women who experienced sPTB and those in the control group. These differences were most pronounced in the initial stages of pregnancy, suggesting that early inflammatory events might play a critical role in predisposing women to sPTB. However, these distinctions gradually diminished as pregnancy progressed into the 2nd and 3rd trimesters. This finding indicates that while systemic inflammation and immune dysregulation are more evident in the early stages of pregnancy for women at risk of sPTB, these inflammatory markers tend to normalize or become less distinct over time. Such normalization could reflect compensatory mechanisms or the progression of underlying inflammatory processes that eventually culminate in preterm labor.

The observation that differences in PLR and LMR are most apparent in the 1st trimester underscores the importance of early detection and intervention strategies aimed at mitigating inflammatory risks. By identifying women with elevated PLR or reduced LMR early in pregnancy, healthcare providers can implement targeted anti-inflammatory therapies or closer monitoring to potentially prevent the onset of sPTB. Moreover, understanding the dynamics of these ratios throughout pregnancy can inform future research on the temporal patterns of inflammatory responses and their implications for maternal and neonatal health. Our findings suggest that while PLR and LMR offer valuable insights into the early inflammatory milieu associated with sPTB, their utility may diminish as pregnancy advances, highlighting the need for longitudinal studies to fully elucidate these relationships. This deeper exploration of PLR and LMR not only aligns with our study objectives but also contributes to a more nuanced understanding of the inflammatory nature of sPTB, ultimately guiding more effective clinical management strategies.

Our findings on the dynamic changes in complete blood count parameters, particularly WBC, monocytes, PLR, and LMR, offer significant potential for both diagnostic and therapeutic applications in managing sPTB. The consistent elevation of WBC and monocyte levels throughout pregnancy in women who experienced sPTB, compared to term controls, underscores their role as markers of ongoing systemic inflammation. These parameters not only reflect heightened inflammatory activity but also provide an opportunity for early intervention. By identifying women with elevated WBC and monocyte counts early in pregnancy, healthcare providers can implement targeted anti-inflammatory therapies or closer monitoring, potentially preventing preterm labor. Furthermore, our study revealed that differences in PLR and LMR were most pronounced during the 1st trimester, suggesting these ratios could serve as early warning signs of increased inflammatory risk. Although these distinctions diminish as pregnancy progresses, their early detection offers a critical window for mitigating inflammatory risks through personalized interventions.

In this study, we reported for the 1st time the changes in routine blood parameters in sPTB during pregnancy. However, larger sample sizes from multiple hospitals could confirm the results in future studies. Meanwhile, prospective studies, not only retrospective studies, can also be conducted to further confirm the results. Although we controlled for age and delivery date in the selection of the control group and further controlled for age and gestational age during the analysis, there may still be potential confounding factors, such as potential biases in data extraction or unmeasured confounders that could lead to bias.

## 5. Conclusions

In conclusion, the current findings demonstrated that blood inflammation- and infection-related parameters, such as WBC count and monocyte count, changed significantly during pregnancy in women with sPTB. Collectively, integrating these blood parameters into routine prenatal care could enhance the predictive accuracy for sPTB, enabling timely and effective management strategies to improve maternal and neonatal outcomes. Future research should focus on validating these findings in larger cohorts and exploring their utility in guiding therapeutic decisions.

## Author contributions

**Conceptualization:** Wen Ai.

**Data curation:** Manhua Zhen, Yanfei Zeng, Chenggang Yang.

**Formal analysis:** Manhua Zhen, Yuhua Song.

**Funding acquisition:** Wen Ai.

**Investigation:** Chenggang Yang.

**Methodology:** Manhua Zhen, Yanfei Zeng, Yuhua Song, Chenggang Yang.

**Project administration:** Wen Ai.

**Resources:** Yanfei Zeng, Chenggang Yang, Wen Ai.

**Software:** Manhua Zhen.

**Supervision:** Wen Ai.

**Validation:** Yuhua Song, Wen Ai.

**Visualization:** Wen Ai.

**Writing – original draft:** Manhua Zhen, Yanfei Zeng.

**Writing – review & editing:** Manhua Zhen, Yanfei Zeng, Yuhua Song, Chenggang Yang, Wen Ai.
